# Digital therapeutics for cognitive impairments associated with schizophrenia: our opinion

**DOI:** 10.3389/fpsyt.2025.1535309

**Published:** 2025-04-17

**Authors:** Shengtao Sun, Chongyang Li, Xueguang Xie, Xianglong Wan, Tiange Liu, Danyang Li, Dingna Duan, Hao Yu, Dong Wen

**Affiliations:** ^1^ School of Information Science and Engineering, Yanshan University, Qinhuangdao, China; ^2^ School of Intelligence Science and Technology, University of Science and Technology Beijing, Beijing, China; ^3^ The Key Laboratory for Brain Computer Intelligence and Digital Therapy of Hebei Province, University of Science and Technology Beijing, Beijing, China; ^4^ Sports Department, University of Science and Technology Beijing, Beijing, China

**Keywords:** schizophrenia, digital therapeutics, cognitive impairment, VR, BCI, EEG analysis, cross-subject, cross-task

## 1 Introduction

Schizophrenia is a psychotic disorder characterized by hallucinations, delusions, disorganized thought and behavior, and psychomotor abnormalities. Cognitive deficits are core features, contributing to long-term morbidity and poor functional outcomes (1). Thus, addressing cognitive deficits is a top research priority. However, the effectiveness of treatments for cognitive impairment associated with schizophrenia (CIAS) remains uncertain. Research suggests that atypical antipsychotics generally have a beneficial effect on neurocognitive deficits (2, 3). However, many atypical antipsychotics are associated with metabolic side effects, such as weight gain, diabetes, and elevated cholesterol or triglyceride levels, which can potentially exacerbate cognitive impairments (4). Cariprazine offers better cognitive improvement in schizophrenia compared to atypical antipsychotics, but it also has some side effects such as akathisia, extrapyramidal symptoms, weight gain, etc (5). Therefore, It is necessary for clinicians and researchers to seek safer and more effective treatment options. Non-pharmacological cognitive therapeutics are primarily represented by traditional cognitive training. Studies have shown that interventions such as metacognitive training (6), social cognition and interaction training (SCIT) or training in affect recognition (7), and Computerized cognitive remediation therapy (8) effectively enhance cognitive function, social interaction, or working memory in patients with schizophrenia. While these approaches offer benefits, they are also limited by factors such as the requirements for professionals in cognitive training and physical training environments.

Recent research has explored using digital technology combined with traditional cognitive training to address CIAS. This approach replaces traditional training with digital systems, including PC applications, mobile apps, and immersive virtual reality (VR) environments, which have been shown to improve cognitive abilities. However, more quantitative methods are needed to evaluate the effectiveness of these interventions and adjust training programs. Given the non-invasive, convenient, and high-temporal-resolution nature of electroencephalogram (EEG) signals, researchers often use them to assess cognitive functions in various brain-computer interface (BCI) tasks (9). EEG signal analysis has been employed to evaluate the effectiveness of spatial memory training using VR software (10). However, significant variability in EEG signals across individuals and tasks diminishes the generalizability of EEG analysis algorithms (11). To address these challenges, researchers have developed cross-subject EEG signal analysis methods to enhance model generalization across different individuals. For instance, the FMLAN framework (12) has been applied to cross-subject emotion recognition, while the DDA model (13) has been utilized for cross-subject cognitive workload recognition. And others have focused on cross-task EEG signal analysis to enhance model generalization across different tasks (14, 15). Nonetheless, there is a lack of research that simultaneously addresses both cross-subject and cross-task EEG signal analysis in cognitive assessment.

Current digital therapies for treating CIAS have several limitations, such as monotonous scenarios and low immersion, which lead to decreased patient adherence and retention rates. This study reviews the literature on digital therapeutics for CIAS, examines the challenges, and offers recommendations for advancement from the perspective of computer science and technology. Stakeholders benefiting from these advancements include clinicians (improved diagnostic tools), patients (better engagement and outcomes), and policymakers (cost-effectiveness and scalability).

## 2 Current status of digital therapeutics for CIAS

Research shows that digital therapeutics can address the unique needs of schizophrenia patients, such as enhancing social skills (16), and have significant potential to improve outcomes in these patients (17). Additionally, digital therapeutics may improve visual attention and logical memory in individuals with schizophrenia during the early stages of the disorder (18). Active video games can enhance prefrontal activity in schizophrenia patients, facilitating their cognitive rehabilitation (19). These digital intervention methods address the limitations of traditional cognitive training and demonstrate enhanced therapeutic potential, such as increasing patient engagement, improving social skills, and enhancing attention. Nonetheless, existing methods often lack sufficient immersion, and assessments are primarily based on traditional neuropsychological tests, which need further refinement. The rapid advancement of VR offers immersive experiences and has considerable potential in the field of cognitive training (20). Meanwhile, as physiological signals, EEG are less influenced by subjective factors, making them a robust quantitative foundation for evaluating the efficacy of cognitive training therapeutics.

### 2.1 Advances in VR intervention

Recently, there has been increasing research interest in utilizing VR training for the development of various skills, including cognitive functions. Kamari et al. demonstrated that VR is an effective tool for enhancing cognitive abilities (20). Xie et al. highlighted the great potential of BCI-VR technology in effectively improving individuals’ executive function (21). The VR serious game training have significantly improved working memory and executive function in CIAS (22). Moreover, VR-SCIT has proven to be more effective than traditional SCIT, leading to improvements in social functioning and emotion perception in patients with schizophrenia (23). Review demonstrated that fully immersive VR can serve as a valuable cognitive rehabilitation intervention for mental illnesses, including schizophrenia (24), with effective clinical feasibility outcomes (25).

These findings collectively highlight the potential of VR in enhancing cognitive functions, such as working memory, executive function, social functioning, and emotion perception in individuals with schizophrenia. The immersive nature of VR can boost patient engagement and treatment adherence (23). It is important to note that VR games may induce cybersickness in patients during gameplay. Therefore, both software and hardware developers should focus on reducing sensory conflicts within the game to mitigate this issue (26). Current VR use for enhancing cognitive function in schizophrenia has several limitations. The VR scenarios often lack diversity, with repetitive tasks like object sorting or navigation exercises, which reduces cognitive stimulation. Moreover, monotonous environments, such as generic virtual rooms or simplistic landscapes, can lead to decreased patient engagement and motivation.

Another significant issue is the insufficient personalization of VR scenes. For example, patients may face tasks in environments that do not reflect their daily challenges, such as navigating a virtual supermarket or interacting with avatars in unrealistic settings. These generic setups fail to replicate the complexities and nuances of real-world situations, limiting the relevance of the training. VR scenarios currently used in research often lack diversity and personalized reproduction of real scenes, underscoring the need for more innovative and adaptable VR designs to create meaningful, context-specific interventions tailored to individual needs.

### 2.2 Advances in BCI cognitive evaluation: cross-subject and cross-task EEG analysis

In studies investigating digital therapeutics for CIAS, cognitive level assessment mainly uses traditional cognitive assessment methods, such as rating scale. These conventional approaches often rely on subjective judgment, making participants vulnerable to environmental influences, which can lead to errors and diminish the validity and reliability of the results. In contrast, EEG signals, due to their non-invasive nature and the effectiveness of analysis techniques in filtering out external noise, offer a more objective and precise assessment. This technological advancement is propelling cognitive assessment into a new dimension. Wen et al. explored a feature extraction method for analyzing EEG signals to assess spatial cognitive abilities (10), offering an innovative and scientifically robust approach to evaluating patients’ cognitive abilities. However, the variability of EEG signals between individuals and tasks can impact the generalizability of EEG analysis algorithms. To address this issue, the EEG signal analysis algorithm is being explored from two perspectives: cross-subject and cross-task.

1) Cross-subject EEG signal analysis: This approach aims to address individual differences in EEG data, thereby improving the generalization performance of models. The FMLAN framework, based on multiple sub-networks and a Fine-grained Alignment Module, has significantly advanced cross-subject EEG emotion recognition (12). Similarly, the DDA model, incorporating EEG feature extraction, label classification, feature distribution alignment, and domain discrimination, has shown strong performance in cross-subject EEG classification (13).
2) Cross-task EEG signal analysis: This method seeks to manage differences between tasks. CTNAS-EEG framework can automatically design the network structure across tasks and improve the recognition accuracy of EEG signals (14). Furthermore, SCDA model achieved the highest average EEG-based mental workload cross-task classification accuracy (75.39% ± 9.56% on experiment data, 90.98% ± 9.36% on the COG-BCI public dataset) (15).

Research on cross-subject and cross-task EEG signal analysis is enhancing generalization capabilities, offering new insights into cognitive evaluation and optimizing training strategies in CIAS. However, integrating both cross-subject and cross-task analysis in EEG-based cognitive evaluation remains a challenge, especially in developing a generalized model to predict cognitive performance across diverse patients and tasks, requiring further exploration. In the future, cross-subject and cross-task EEG analysis could benefit from integrating EEG with AI models or expanding datasets to include underrepresented populations.

## 3 Trends in digital therapeutics for CIAS

**Figure 1:**
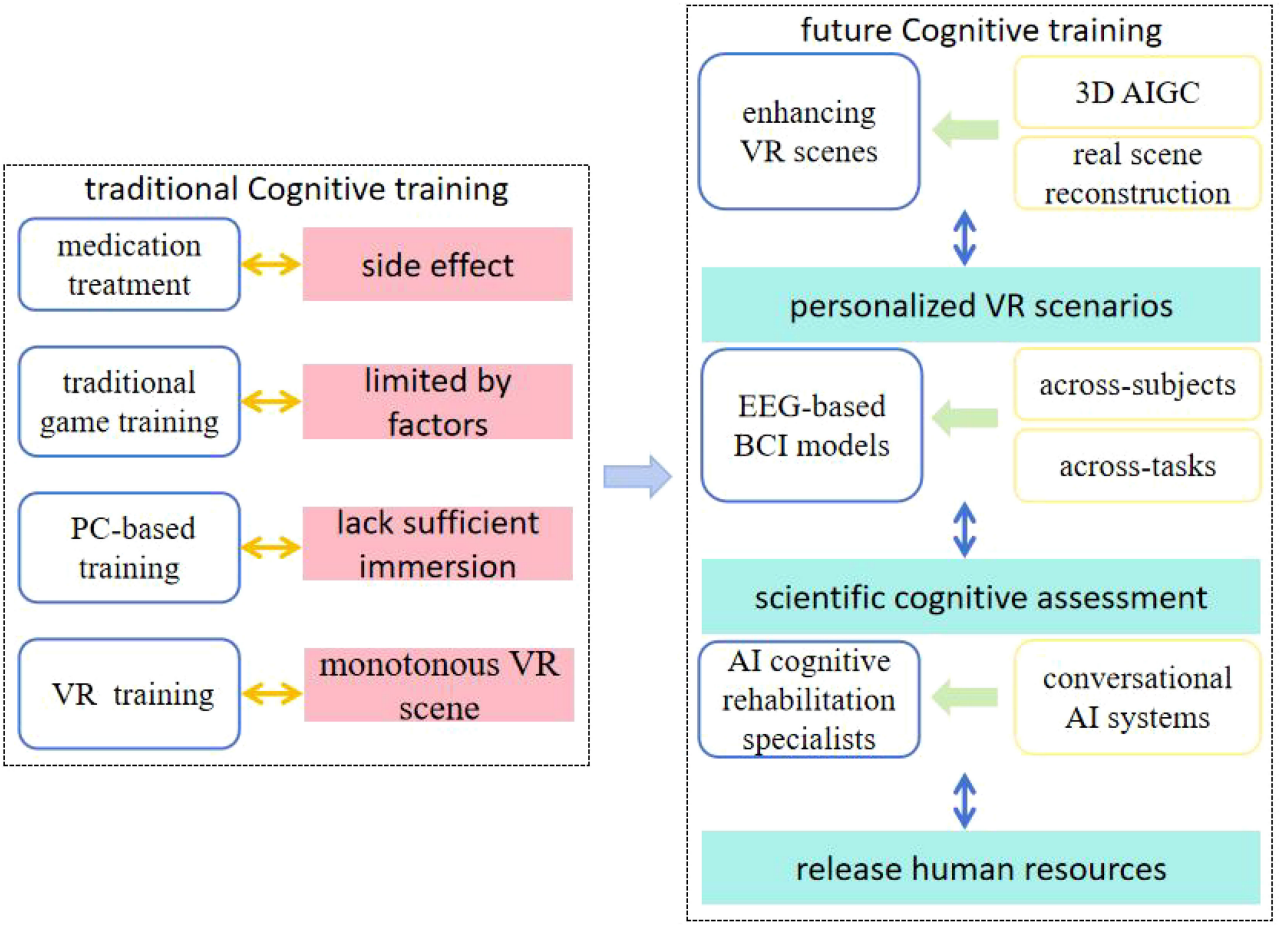
Conceptual framework of digital therapeutics in future CIAS.

The previous section explored digital therapeutics for CIAS, showing promising results but requiring further investigation. This section looks at future pathways and emerging trends in digital therapeutics for schizophrenia, focusing on how innovations like VR, BCI, and AI could offer new solutions for this complex disorder. Here is the conceptual map for the future (**Figure 1**).

1) Leveraging VR for immersive and personalized training scenarios

Schizophrenia patients often show low treatment engagement. Personalized VR scenarios can stimulate patients’ interest, leading to better treatment outcomes and a more positive recovery outlook. The rapid advancement of AI-generated content (AIGC) has enabled the generation of VR content, including 3D objects and 360-degree images, based on textual prompts, sketches, images, and other inputs (27). The 3D Gaussian splatting algorithm allows for fast real-world scene 3D reconstruction from multi-view images, making it possible to rapidly generate interactive and realistic VR environments (28, 29). Using 3D reconstruction and AIGC, along with user language text, images, and videos, to craft personalized and engaging VR scenarios for CIAS training will significantly enhance patient engagement, making the treatment process both efficient and enjoyable. For example, generating diverse VR scenarios, such as space and underwater, or reconstructing real scenes familiar to patients, such as their home, community, and streets, will more effectively support and enhance the training process.

2) Developing an EEG-based BCI cognitive assessment model to optimize training strategies

Future research should focus on developing universal EEG-based quantitative cognitive assessment methods for cross-subject and cross-task EEG analysis (30), which will provide a scientific framework for digital therapeutics in CIAS. Additionally, integrating BCI technology for real-time data collection and analysis during training can enable immediate feedback for adjusting training strategy, facilitating an adaptive, personalized intervention strategy. Moreover, the development of portable all-in-one BCI-VR systems with cloud service, could reduce the need for specialized user equipment. This would significantly improve the accessibility of cognitive rehabilitation for schizophrenia patients, particularly in low-resource settings.

3) Developing AI cognitive rehabilitation specialists to enhance the efficiency and accessibility of rehabilitation services

As AI technology evolves, AI conversational systems, such as GPT (31) and LLaMA (32), have become more refined. Integrating these systems with cognitive rehabilitation and developing AI rehabilitation specialists can replace traditional professionals, reduce healthcare costs, and address workforce shortages. This innovative approach will improve the efficiency and reach of rehabilitation services, providing personalized cognitive rehabilitation plans for each patient with CIAS and ensuring precision and customization throughout the rehabilitation process. Imagine that each patient will have their own individual AI cognitive rehabilitation specialist who will remind or cooperate with the patient for cognitive rehabilitation training every day, and automatically develop a more reasonable training plan for the next session based on the patient’s training situation, which is very helpful for the patient’s cognitive rehabilitation. However, ethical concerns, such as potential bias in AI-driven systems, must be addressed. Regular audits and transparent algorithms can be implemented to ensure reliability and mitigate these risks.

## 4 Conclusion

This paper explores digital therapeutics for cognitive impairments associated with schizophrenia, highlighting their flexibility and adaptability in cognitive training compared to traditional methods. Besides, the use of VR and EEG analysis can further enhance the effectiveness of cognitive training and assessment. However, current VR scenes for cognitive training always lack diversity, personalization, and realistic scenes. And quantitative EEG-based cognitive assessments remain constrained in their applicability to cross-subject and cross-task scenarios. Therefore, we studied the emerging directions in digital therapeutics for CIAS from the perspective of computer science and technology, focusing on three key areas: 1) enhancing the diversity of VR scenes through real scene reconstruction and 3D AIGC, 2) advancing cross-subject and cross-task cognitive assessment methods using EEG-based BCI models, and 3) developing AI-driven cognitive rehabilitation specialists by integrating conversational AI systems to reduce human labor. We believe that the application of these innovative technologies will not only significantly improve treatment outcomes but also provide patients with a more personalized and precise therapeutic experience for CIAS, while making treatments more accessible and reducing healthcare inequalities.

## References

[1] McCutcheon RA Keefe RS McGuire PK. Cognitive impairment in schizophrenia: aetiology, pathophysiology, and treatment. Mol Psychiatry. (2023) 28:1902–18. doi: 10.1038/s41380-023-01949-9 PMC10575791 36690793

[2] Hou Y Xie J Yuan Y Cheng Z Han X Yang L. Neurocognitive effects of atypical antipsychotics in patients with first-episode schizophrenia. Nordic J Psychiatry. (2020) 74:594–601. doi: 10.1080/08039488.2020.1771767 32496921

[3] Baldez DP Biazus TB Rabelo-da-Ponte FD Nogaro GP Martins DS Kunz M. The effect of antipsychotics on the cognitive performance of individuals with psychotic disorders: network meta-analyses of randomized controlled trials. Neurosci Biobehav Rev. (2021) 126:265–75. doi: 10.1016/j.neubiorev.2021.03.028 33812977

[4] MacKenzie NE Kowalchuk C Agarwal SM Costa-Dookhan KA Caravaggio F Gerretsen P. Antipsychotics, metabolic adverse effects, and cognitive function in schizophrenia. Front Psychiatry. (2018) 9:622. doi: 10.3389/fpsyt.2018.00622 30568606 PMC6290646

[5] Laszlovszky I Barabássy Á. Németh G. Cariprazine, a broad-spectrum antipsychotic for the treatment of schizophrenia: pharmacology, efficacy, and safety. Adv Ther. (2021) 38:3652–73. doi: 10.1007/s12325-021-01797-5 PMC8279990 34091867

[6] Hotte-Meunier A Penney D Mendelson D Thibaudeau É. Moritz S Lepage M. Effects of metacognitive training (MCT) on social cognition for schizophrenia spectrum and related psychotic disorders: a systematic review and meta-analysis. psychol Med. (2024) 54:914–20. doi: 10.1017/S0033291723002611 37772399

[7] Lahera G Reboreda A Vallespí A Vidal C López V Aznar A. Social cognition and interaction training (SCIT) versus training in affect recognition (TAR) in patients with schizophrenia: a randomized controlled trial. J Psychiatr Res. (2021) 142:101–9. doi: 10.1016/j.jpsychires.2021.07.029 34332374

[8] Yamanushi A Shimada T Koizumi A Kobayashi M. Effect of computer-assisted cognitive remediation therapy on cognition among patients with schizophrenia: A pilot randomized controlled trial. Biomedicines. (2024) 12:1498. doi: 10.20944/preprints202406.0414.v1 39062072 PMC11274551

[9] Wen D Yuan J Zhou Y Xu J Song H Liu Y. The eeg signal analysis for spatial cognitive ability evaluation based on multivariate permutation conditional mutual information-multi-spectral image. IEEE Trans Neural Syst Rehabil Eng. (2020) 28:2113–22. doi: 10.1109/TNSRE.2020.3018959 32833638

[10] Wen D Liang B Li J Wu L Wan X Dong X. Feature extraction method of EEG signals evaluating spatial cognition of community elderly with permutation conditional mutual information common space model. IEEE Trans Neural Syst Rehabil Eng. (2023) 31:2370–80. doi: 10.1109/TNSRE.2023.3273119 37141070

[11] Wu D Xu Y Lu B. Transfer learning for eeg-based brain–computer interfaces: a review of progress made since 2016. IEEE Trans Cognit Dev Syst. (2022) 14:4–19. doi: 10.1109/TCDS.2020.3007453

[12] Yu P He X Li H Dou H Tan Y Wu H. FMLAN: a novel framework for cross-subject and cross-session EEG emotion recognition. Biomed Signal Process Control. (2025) 100:106912. doi: 10.1016/j.bspc.2024.106912

[13] Zhou Y Wang P Gong P Wei F Wen X Wu X. Cross-subject cognitive workload recognition based on eeg and deep domain adaptation. IEEE Trans Instrum Meas. (2023) 72:1–12. doi: 10.1109/TIM.2023.3276515 37323850

[14] Duan Y Wang Z Li Y Tang J Wang Y Lin C. Cross task neural architecture search for eeg signal recognition. Neurocomputing. (2023) 545:126260. doi: 10.1016/j.neucom.2023.126260

[15] Wang T Ke Y Huang Y He F Zhong W Liu S. Using semi-supervised domain adaptation to enhance EEG-based cross-task mental workload classification performance. IEEE J Biomed Health Inf. (2024) 28:7032–9. doi: 10.1109/JBHI.2024.3452410 39213268

[16] Campellone T Carretta C Davidson C Lakhan S Sand M. Assessing the unmet clinical need and opportunity for digital therapeutic intervention in schizophrenia: perspective from people with schizophrenia. CNS Spectrums. (2023) 28:263–3. doi: 10.1017/S1092852923002146

[17] Fulford D Marsch LA Pratap A. Prescription digital therapeutics: An emerging treatment option for negative symptoms in schizophrenia. Biol Psychiatry. (2024) 96:659–65. doi: 10.1016/j.biopsych.2024.06.026 PMC11410508 38960019

[18] Fernandez-Gonzalo S Turon M Jodar M Pousa E Hernandez Rambla C García R. A new computerized cognitive and social cognition training specifically designed for patients with schizophrenia/schizoaffective disorder in early stages of illness: a pilot study. Psychiatry Res. (2015) 228:501–9. doi: 10.1016/j.psychres.2015.06.007 26163731

[19] Shimizu N Umemura T Matsunaga M Hirai T. An interactive sports video game as an intervention for rehabilitation of community-living patients with schizophrenia: a controlled, single-blind, crossover study. PloS One. (2017) 12:e187480. doi: 10.1371/journal.pone.0187480 PMC5683619 29131826

[20] Kamari M Siqueira V Bakare J Sebastião E. Virtual reality technology for physical and cognitive function rehabilitation in people with multiple sclerosis. Rehabil Res Pract. (2024) 2024:2020263. doi: 10.1155/2024/2020263 39355707 PMC11444799

[21] Xie X Shi R Yu H Wan X Liu T Duan D. Executive function rehabilitation and evaluation based on brain-computer interface and virtual reality: our opinion. Front Neurosci. (2024) 18:1377097. doi: 10.3389/fnins.2024.1377097 38808030 PMC11130371

[22] Wang X Kou X Meng X Yu J. Effects of a virtual reality serious game training program on the cognitive function of people diagnosed with schizophrenia: a randomized controlled trial. Front Psychiatry. (2022) 13:952828. doi: 10.3389/fpsyt.2022.952828 35911215 PMC9334918

[23] Shen Z Liu M Wu Y Lin Q Wang Y. Virtual-reality-based social cognition and interaction training for patients with schizophrenia: a preliminary efficacy study. Front Psychiatry. (2022) 13:1022278. doi: 10.3389/fpsyt.2022.1022278 36465308 PMC9714325

[24] Jahn FS Skovbye M Obenhausen K Jespersen AE Miskowiak KW. Cognitive training with fully immersive virtual reality in patients with neurological and psychiatric disorders: A systematic review of randomized controlled trials. Psychiatry Res. (2021) 300:113928. doi: 10.1016/j.psychres.2021.113928 33857847

[25] Perra A Riccardo CL De Lorenzo V De Marco E Di Natale L Kurotschka PK. Fully immersive virtual reality-based cognitive remediation for adults with psychosocial disabilities: a systematic sco** review of methods intervention gaps and meta-analysis of published effectiveness studies. Int J Environ Res Public Health. (2023) 20:1527. doi: 10.3390/ijerph20021527 36674283 PMC9864668

[26] Shafer DM Carbonara CP Korpi MF. Factors affecting enjoyment of virtual reality games: a comparison involving consumer-grade virtual reality technology. Games Health J. (2019) 8:15–23. doi: 10.1089/g4h.2017.0190 30199273

[27] Cai Z Mueller M Birkl R Wofk D Tseng SY Cheng J. L-MAGIC: language model assisted generation of images with coherence. In: Proceedings of the IEEE/CVF conference on computer vision and pattern recognition (2024). p. 7049–7058. doi: 10.1109/CVPR52733.2024.00673

[28] Kerbl B Kopanas G Leimkühler T Drettakis G. 3D gaussian splatting for real-time radiance field rendering. ACM Trans Graph. (2023) 42:139–1. doi: 10.1145/3592433

[29] Jiang Y Yu C Xie TY Li X Feng YT Wang HM. VR-GS: a physical dynamics-aware interactive gaussian splatting system in virtual reality. In ACM SIGGRAPH 2024 Conference Papers (SIGGRAPH '24). Association for Computing Machinery, New York, NY, USA. (2024), 78. doi: 10.1145/3641519

[30] Jiang WB Zhao LM Lu BL. Large brain model for learning generic representations with tremendous EEG data in BCI. In: The twelfth international conference on learning representations (2024) arxiv:2405.18765. doi: 10.48550/arXiv.2405.18765

[31] Achiam J Adler S Agarwal S Ahmad L Akkaya I Aleman FL. Gpt-4 technical report. arxiv. (2023) arXiv:2303.08774. doi: 10.48550/arXiv.2303.08774

[32] Touvron H Lavril T Izacard G Martinet X Lachaux MA Lacroix T. Llama: Open and efficient foundation language models. arxiv. (2023) arXiv:2302.13971. doi: 10.48550/arXiv.2307.09288

